# Phobic postural vertigo treated with autogenic training: a case report

**DOI:** 10.1186/1757-1626-1-189

**Published:** 2008-09-30

**Authors:** Fumiyuki Goto, Kimiko Nakai, Takanobu Kunihiro, Kaoru Ogawa

**Affiliations:** 1 Department of Otorhinolaryngology, Hino Municipal Hospital, Tamadaira 4-3-1, Hino-shi, Tokyo 191-0062, Japan; 2 Department of Otolaryngology, Keio University School of Medicine, 35 Shinanomachi, Shinjuku-ku, Tokyo 160-8520, Japan

## Abstract

**Background:**

Patients suffering from dizziness due to vertigo are commonly encountered in the department of otolaryngology. If various clinical examinations do not reveal any objective findings, then the patients are referred to the department of internal medicine or psychiatry. In many cases, the diagnosis is psychological dizziness. Phobic postural vertigo, which was first reported by Brandt T et al in 1994, is supposed to be a type of psychological dizziness. The diagnosis is based on 6 characteristics proposed by Brandt et al. Patients are usually treated with conventional medical therapy, but some cases may be refractory to such a therapy. Psychotherapy is recommended in some cases; however, psychotherapy including autogenic training, which can be used for general relaxation, is not widely accepted. This paper describes the successful administration of autogenic training in a patient suffering from phobic postural vertigo.

**Case presentation:**

We present a case of a patient who suffered from phobic postural vertigo. A 37-year-old female complained of dizziness. She had started experiencing dizziness almost 3 years She was intractable to many sort of conventional therapy. In the end, her symptom disappeared after introduction of autogenic training.

**Conclusion:**

Autogenic training can be a viable and acceptable treatment option for phobic postural vertigo patients who fail to respond to other therapies. This case emphasizes the importance of autogenic training as a method to control symptom of phobic postural vertigo.

## Background

Patients suffering from dizziness are commonly encountered at the otologist in the department of otorhinolaryngology. In the absence of any organic abnormality, such patients are diagnosed with psychogenic dizziness and are often referred to the department of psychosomatic medicine or to a psychologist, because dizziness and vertigo are complaints common to various psychiatric conditions like major depression, somatoform disorder, and anxiety disorders. A representative etiology is phobic postural vertigo (PPV), which was first proposed by Brandt T et al in 1994 [1]. The diagnosis of PPV is based on 6 characteristics proposed by this group. The key features for correct diagnosis are spontaneous (sometimes stimulus-induced) postural vertigo and unsteadiness in maintaining an upright position and walking; the correct diagnosis is not based on anxiety but on subjective dizziness itself. The correct diagnosis of PPV is important for better prognosis. These patients often have an obsessive personality. Psychotherapy including autogenic training (AT) and cognitive behavior therapy (CBT), which can be used for general relaxation and to treat disturbed emotions, is a good treatment option. However, there are no reports on the application of AT to patients with PPV. The present paper describes the successful administration of AT to a patient suffering from PPV intractable to several conventional therapies. Written informed consent was obtained from the patient for the presentation of this case.

## Case presentation

A 37-year-old female complained of dizziness. She had started experiencing dizziness 3 years ago, following the infertility treatment that she had received. She experienced dizziness following an injection of human menopausal gonadotropin administered by a gynecologist and a visit psychologist. In addition to her dizziness, she also suffered from insomnia, tinnitus, and anxiety. Therefore, she was referred to a psychologist. However, the treatment of tranquilizers such as benzodiazepines and antidepressants such as serotonin selective re-uptake inhibitors (SSRIs) failed to cure the dizziness and only slightly improved her insomnia. She was therefore referred to our department for further examination and treatment. She expressed her dizziness as an event wherein she experienced frequent paroxysmal earthquakes occurring within seconds. The frequency of such episodes had recently increased to once every 5 minutes. She felt stable while doing her household tasks and she had never fallen. Audio-vestibular examination, including pure tone audiometry, posturography, and head MRI, revealed no abnormal findings. Her blood examination findings were normal; there was no spontaneous or evoked nystagmus. However, the peripheral part of her hand and foot often became pale due to poor peripheral circulation, an observation similar to Raynaud's phenomenon. She also experienced chronic headache and insomnia, whereby she woke up every 2 hours during the night. The results of the psychological examination were as follows: Self-rating Depression Scale (SDS), 47; Japanese version of the Cornell Medical Index (CMI), III; Manifest Anxiety Scale (MAS), 27; and Maudsley Obsessional-Compulsive Inventory (MOCI [2]), 9. MAS indicated a high level of anxiety. We deduced that her dizziness was due to psychosomatic reasons together with poor peripheral circulation. We prescribed setiptiline maleate and an additional herbal medicine, which is known to improve peripheral circulation. Within 2 weeks her symptoms slightly improved and the level of dizziness reduced to less than one third. However she didn't want to keep taking these drugs, since she want to have a baby. No abnormality was reported in any physical examination, including posturography. We diagnosed the patient's condition as phobic postural vertigo. Although she often experienced palpitations, cardiological examination reported no abnormal findings. These results indicate the existence of autonomic dysfunction due to psychological stress, including anxiety. We decided to focus on treating the patient's anxiety and the supposed autonomic dysfunction. After 1 month following the patient's first visit, AT was introduced by a clinical psychologist so as to ease her mental stress. The psychotherapy consisted of one 45-minute session every 3 weeks. The first session began with a brief introduction to the general background information about the cognitive approach, after which the patient was instructed how to perform AT. Thereafter, the patient performed AT in a relaxed sitting position on a chair for 10 minutes 3 times a day. No self-monitoring was advised. The patient was instructed to carry out slow and deep abdominal breathing at the beginning of AT and regular breathing during AT. She diligently and regularly continued this AT routine 3 times a day at her home, according to a written timetable. She learned all 6 standard formulas of AT in 6 psychotherapy sessions. Astonishingly, after the introduction of AT, her mood stabilized and her dizziness, insomnia, and headache disappeared in a few weeks. The dose of clotiazepam was reduced to 5 mg once a day. No additional treatment was administered. At 6- and 9-month follow-ups, the patient was free from dizziness, insomnia, and headache.

## Conclusion

The diagnosis of PPV is based on 6 characteristics proposed by Brandt et al. The diagnostic criteria [1] are as follows: (1) dizziness and subjective disturbance of balance while standing or walking despite normal clinical balance tests; (2) fluctuating unsteadiness for seconds to minutes, or momentary perceptions of illusory body perturbations; (3) usually a perceptual stimulus or social situation as a provoking factor with a tendency for rapid conditioning, generalization, and avoidance behavior; (4) anxiety and vegetative symptoms during or after vertigo; (5) obsessive-compulsive personality type, labile affect, or mild depression; and (6) onset frequently after a period of emotional stress, serious illness, or a vestibular disorder. Patients sometimes exhibit anxiety reactions. The different possible treatments for PPV include pharmacological treatment and psychotherapy. In this case, we clearly showed that patients with PPV that is intractable to many types of drugs can be alleviated by AT, which is a type of psychotherapy.

AT was developed by the German psychiatrist Johannes Schultz and can be achieved by daily self-training sessions of 10 to 15 minutes [3]. AT is a technique for influencing one's autonomic nervous system and it can be used to alleviate many stress-induced psychosomatic disorders. Schultz emphasized parallelism between AT, yoga and meditation. AT has been widely applied as a relaxation technique and has been viewed as a highly effective method for controlling pain and reducing drug dependence substantially [4].

**Figure 1:**
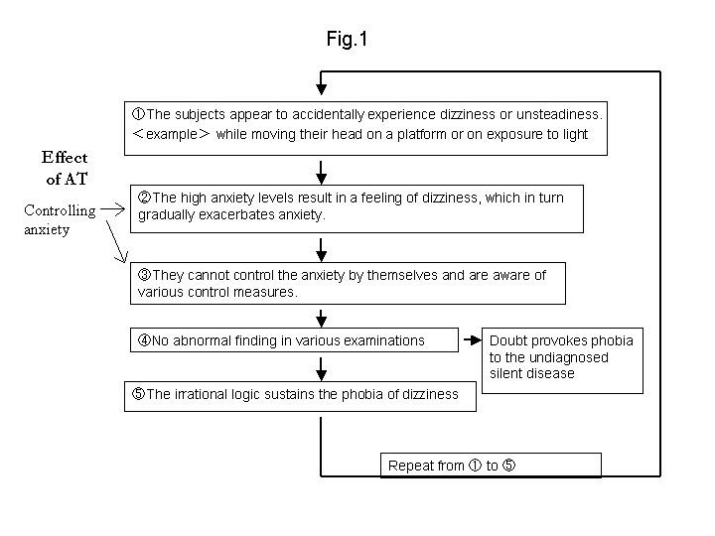
The vicious circle of phobic postural vertigo (PPV) and the effect of autogenic training.

We used a psychological approach to treat this patient because her symptoms were closely related to her anxiety and autogenic dysfunction and were refractory to conventional therapy. We speculate a vicious circle of PPV and the effect of AT, as shown in Fig. 1. We now assume patients who have PPV with the following characteristics to be candidates for psychotherapy: (1) patients with dizziness, which is intractable to conventional therapy; (2) patients with insomnia; (3) patients with a high anxiety level; (4) patients that have a variety of complaints in addition to dizziness; and (5) patients in whom vertigo is believed to be triggered by stress. However, AT is not recommended for patients with low-level anxiety, those with little motivation, or those who lack the intellectual capacity to understand and perform AT [5,6]. AT can be a viable and acceptable treatment option for a patient with PPV refractory to other therapies.

## Authors' contributions

All authors read and approved the final manuscript. FG and KN participated in the treatment of the patient and drafted the manuscript. TK and KO provided instructions and advice on the treatment strategy.

## Consent

Written informed consent was obtained from the patient for publication of this case report and accompanying images. A copy of the written consent is available for review by the Editor-in-Chief of this journal.
